# Co-existing cerebrovascular atherosclerosis predicts subsequent vascular event: a multi-contrast cardiovascular magnetic resonance imaging study

**DOI:** 10.1186/s12968-019-0596-6

**Published:** 2020-01-13

**Authors:** Jin Li, Dongye Li, Dandan Yang, Ran Huo, Xiaoyi Chen, Yilan Xu, Wei Dai, Dan Zhou, Xihai Zhao

**Affiliations:** 1 Department of Radiology, The Affiliated BenQ Hospital of Nanjing Medical University, Nanjing, China; 2 Center for Biomedical Imaging Research, Department of Biomedical Engineering, Tsinghua University School of Medicine, Haidian District, Beijing, 100084 China; 3 Department of Radiology, Sun Yat-Sen Memorial Hospital, Sun Yat-Sen University, Guangzhou, China; 4 Beijing Institute of Brain Disorders, Capital Medical University, Beijing, China; 5 Department of Radiology, Peking University Third Hospital, Beijing, China; 6 Department of Radiology, Beijing Geriatric Hospital, Beijing, China; 7 Department of Radiology, Beijing Tsinghua Changgung Hospital, School of Clinical Medicine, Tsinghua University, Beijing, China; 8 Department of Neurology, Fourth Medical Center of Chinese PLA General Hospital, Beijing, China

**Keywords:** Cerebrovascular, Atherosclerosis, Cardiovascular magnetic resonance imaging, Stroke, Acute coronary syndrome

## Abstract

**Background:**

It is still unknown that whether co-existing intracranial stenosis and extracranial carotid vulnerable plaques have higher predictive value for subsequent vascular events. This study aimed to determine the relationship between co-existing extracranial carotid vulnerable plaques and intracranial stenosis and subsequent vascular events utilizing cardiovascular magnetic resonance (CMR) vessel wall imaging.

**Methods:**

Patients who had recent cerebrovascular symptoms in anterior circulation (< 2 weeks) were consecutively enrolled and underwent multi-contrast CMR vessel wall imaging for extracranial carotid arteries and 3D time-of flight CMR angiography for intracranial arteries at baseline. After baseline examination, all patients were followed-up for at least 1 year to determined recurrence of vascular events. The co-existing cerebrovascular atherosclerosis was defined as presence of both intracranial artery stenosis and at least one the following measures of extracranial artery atherosclerosis: plaque, calcification, lipid-rich necrotic core (LRNC), or intraplaque hemorrhage. Univariate and multivariate Cox regressions were used to calculate the hazard ratio (HR) and corresponding 95% confidence interval (CI) of co-existing plaques in predicting subsequent vascular events.

**Results:**

In total, 150 patients (mean age: 61.8 ± 11.9 years; 109 males) were recruited. During the median follow-up time of 12.1 months, 41 (27.3%) patients experienced vascular events. Co-existing intracranial artery stenosis and extracranial carotid plaque (HR, 3.57; 95% CI, 1.63–7.82; *P* = 0.001) and co-existing intracranial artery stenosis and extracranial carotid LRNC (HR, 4.47; 95% CI, 2.15–9.27; *P* < 0.001) were significantly associated with subsequent vascular events, respectively. After adjusted for confounding factors and carotid stenosis, these associations remained statistically significant (HR, 5.12; 95% CI, 1.36–19.24; *P* = 0.016 and HR, 8.12; 95% CI, 2.41–27.31; *P* = 0.001, respectively).

**Conclusions:**

The co-existing cerebrovascular atherosclerotic diseases, particularly co-existing carotid lipid-rich necrotic core and intracranial stenosis, are independent predictors for subsequent vascular events.

## Introduction

It has been shown that vulnerable atherosclerotic plaque is the major cause of ischemic stroke or acute coronary syndrome [1–3]. As a systematic disease, atherosclerotic disease commonly affects multiple vascular beds. The presence of atherosclerotic diseases in multiple vascular beds is usually defined as co-exiting plaques which represent the systemic burden of atherosclerosis [4, 5]. Previous studies demonstrated that co-existing intracranial and extracranial atherosclerotic plaques had stronger predictive value for future vascular events compared with atherosclerosis in single vascular bed [6, 7]. However, the assessment of co-existing plaques in most of previous studies was focused on either the presence of plaques or measuring luminal stenosis. It is still unknown that whether co-existing intracranial stenosis and extracranial carotid vulnerable plaques have higher predictive value for subsequent vascular events. We hypothesized that co-existing carotid vulnerable plaque and intracranial stenosis may have stronger predictive value than each measurement alone for subsequent vascular events.

The co-existing intracranial and extracranial plaques can be characterized by multiple noninvasive imaging modalities, such as computed tomographic angiography (CTA) and cardiovascular magnetic resonance (CMR) angiography (CMRA) in clinical settings. However, both CTA and CMRA techniques could not provide the key compositional features of vulnerable plaques, such as lipid-rich necrotic core (LRNC) and intraplaque hemorrhage (IPH). This is because that these imaging modalities lack of vessel information. In contrast, it is well established that CMR vessel wall imaging is capable of accurately characterizing vulnerable plaque features in carotid arteries validated by histology [8].

This study aimed to determine the relationship between co-existing extracranial carotid vulnerable plaques and intracranial stenosis and subsequent vascular events utilizing CMR vessel wall imaging.

## Methods

### Study population

In this prospective study, patients who had recent cerebrovascular symptoms (ischemic stroke or transient ischemic attack [TIA] in the anterior circulation < 2 weeks) were consecutively enrolled and underwent multi-contrast CMR vessel wall imaging for extracranial carotid arteries and three dimensional time-of-flight (3D-TOF) CMRA for intracranial arteries at baseline. The exclusion criteria are as follows: (1) hemorrhagic stroke; (2) cardiogenic stroke; (3) other vascular diseases including dissection, vasculitis, and moyamoya disease; (4) cerebral tumor; (5) history of radiation therapy in the neck; (6) pregnancy; (7) patients with severe disturbance of consciousness (coma, etc); (8) claustrophobia or any contraindication to CMR examination. Baseline clinical information for all patients including age, sex, body mass index (BMI), history of smoking, diabetes, hypertension, hyperlipidemia, stroke, coronary heart disease, National Institutes of Health Stroke Scale (NIHSS) score, the levels for lipoproteins (high density lipoprotein, low density lipoprotein, total cholesterol, and triglyceride) and the blood pressure was collected from the medical records. The information on treatment of antihypertension, lipid-lowering, anticoagulation and antiplatelet was also recorded during follow-up. The study protocol was approved by institutional review board and written consent form was obtained from each participant.

### Imaging acquisition

At baseline, extracranial carotid artery and intracranial artery CMR imaging was conducted for all the patients on a 3 T CMR scanner (Achieva TX, Philips Healthcare, Best, The Netherlands) with custom-designed 36-channel neurovascular coil. The extracranial carotid arteries were imaged by using multi-contrast vessel wall imaging protocol that includes T1-weighted (T1W), T2-weighted (T2W), 3D-TOF, and magnetization-prepared rapid acquisition gradient echo (MP-RAGE) and intracranial arteries were imaged by acquiring 3D-TOF CMRA. The imaging parameters are detailed in Table 1. In addition, a 3D motion sensitized driven equilibrium rapid gradient echo (3D-MERGE) sequence was acquired for extracranial carotid artery vessel wall imaging with the following parameters: fast field echo, repeat time / echo time 9.2/4.3 ms, flip angle 6°, field of view 20 × 16 × 4 cm^3^; isotropic spatial resolution 0.8 × 0.8 × 0.8 mm^3^. A routine protocol, including T1w, fluid attenuated inversion recovery (FLAIR) and diffusion weighted imaging (DWI) sequences, was used to assess the cerebral infarct during follow-up.

**Table 1:** The parameters for carotid artery and intracranial artery CMR imaging

	CMR imaging parameters				
	Extracranial carotid artery				Intracranial artery
	T1W	T2W	TOF	MP-RAGE	TOF-CMRA
Sequence	TSE	TSE	FFE	FFE	FFE
Black blood	QIR	MDIR	–	–	–
TR, ms	800	4800	20	8.8	25
TE, ms	10	50	4.9	5.3	3.5
Flip angle, deg	90°	90°	20°	15°	20°
FOV, mm	140 × 140	140 × 140	140 × 140	140 × 140	240 × 240
In-plane resolution, mm^2^	0.5 × 0.5	0.5 × 0.5	0.5 × 0.5	0.5 × 0.5	0.7 × 0.7
Slice thickness, mm	2	2	1	1	1.4

*TOF* time-of-flight, *MP-RAGE* Magnetization Prepared Gradient Recalled Echo, *CMRA* cardiovascular magnetic resonance angiography, *TSE* turbo spin echo, *FFE* fast field echo, *QIR* quadruple inversion recovery, *MDIR* multislice double inversion recovery. *TR* repeat time, *TE* echo time, *FOV* field of view, *T1W* T1 weighted, *T2W* T2 weighted

### Follow-up and clinical outcome

After CMR imaging at baseline, all patients were followed-up for at least 1 year. The clinical outcome was defined as any vascular event, such as ischemic stroke, TIA, or acute coronary syndrome. All patients were followed-up by telephone or routine medical outpatient clinic attendance and inquiring whether patients had experienced any vascular event in the past time. If patients did not respond to the follow-up, we will find their medical records and try to contact their relatives for more information about the patient’s condition.

### Image analysis

The CMR images were interpreted by two reviewers with > 3 years’ experience in vascular imaging using custom-designed software of CASCADE (University of Washington, Seattle, Washington, USA) with consensus blinded to clinical information. According to the sharpness and contrast between the vessel wall and the surrounding fat tissues by eyeballing, a 4-point scale was adopted: 1, poor; 2, marginal; 3, good; 4, excellent. The CMR images with image scale ≥2 were interpreted. Carotid plaque was defined as intima-media thickness ≥ 1.5 mm on ultrasound, eccentric wall thickening or having any plaque compositions such as calcification, LRNC, or IPH on CMR. The boundaries of carotid artery lumen and wall were traced manually and the maximum wall thickness (Max WT) which was a representative of plaque burden was measured. For patients with ischemic stroke, the carotid artery which is responsible for the symptoms was considered as index side and included in the statistical analysis. For patients with TIA, the index artery was defined as lesions with high-risk plaque feature, including large LRNC (occupied > 40% of wall area), IPH and fibrous cap rupture, or larger Max WT bilaterally when there was no high-risk feature. The intracranial artery stenosis was measured on 3D-TOF CMRA images, in the following segments of arteries: intracranial internal carotid arteries, anterior cerebral arteries (A1) and middle cerebral arteries (M1). The presence or absence of each plaque composition, such as calcification, LRNC, IPH, was determined by utilizing published criteria [9, 10]. The luminal stenosis was measured using WASID [11] criteria for intracranial arteries and North American Symptomatic Carotid Endarterectomy Trial (NASCET) [12] criteria for extracranial carotid arteries, respectively. The intracranial artery stenosis was categorized into the following categories: (1) < 50%; and (2) ≥50%. The extracranial carotid artery stenosis was categorized into the following categories: (1) < 50%; (2) 50–69%; and (3) ≥70%. The carotid culprit lesion was defined as atherosclerotic plaque with > 50% stenosis or high-risk feature, such as large LRNC (occupied > 40% of wall area), IPH and fibrous cap rupture. For intracranial arteries, the culprit lesion was defined as lesion with > 50% stenosis. The co-existing cerebrovascular atherosclerotic diseases were defined as presence of both intracranial artery stenosis and at least one the following measures of extracranial artery atherosclerosis: plaque, calcification, LRNC, or IPH. The presence or absence of cerebral infarct of anterior circulation was assessed on DWI or FLAIR images during follow-up. The presence or absence of acute coronary syndrome was determined by myocardial enzyme detection or coronary arteriography during follow-up.

### Reproducibility

Twenty patients were randomly selected for testing the intra-observer and inter-observer reproducibility in identifying presence of plaque, calcification, LRNC, IPH and measuring Max WT and stenosis at extracranial carotid artery. The intracranial artery stenosis was also measured for all 20 patients. A time interval of 3 months was set for determining the intra-observer reproducibility to minimize the bias of memory.

### Statistical analysis

The continuous variables were described as mean and standard deviation and the categorical variables were presented as frequency. Baseline clinical characteristics, carotid plaque features and intracranial artery stenosis were compared between patients with and without VEs using independent *t* test, Mann-Whitney U test, or Chi-square when appropriate. In analyzing the predictors of the clinical outcome, univariate and multivariate Cox proportional hazards regression functions were used to calculate hazard ratio (HR) and the corresponding 95% confidence interval (CI) of possible determinants of vascular events, taking the time variable into consideration. The Kaplan-Meier product-limit method was used to estimate cumulative event-free rates in subgroups for graphical display depending on the presence of co-existing plaques. Cohen’s kappa was utilized to determine the inter-observer and intra-observer agreement in identification of presence of carotid plaque, calcification, LRNC and IPH. The intra-class correlation coefficient (ICC) was calculated to assess the inter-observer and intra-observer agreements in quantitatively measuring the intracranial artery stenosis, carotid artery stenosis and Max WT. The *P* value < 0.05 was considered as statistically significant. The statistical analyses were carried out using SPSS (version 19, Statistical Package for the Social Sciences, International Business Machines, Inc., Armonk, New York, USA).

## Results

Of 173 recruited patients, 23 were excluded including 7 with poor image quality, 8 with loss of follow-up and 8 with carotid surgery during follow-up. Among the remaining 150 patients [61.8 ± 11.9 years; 109 (72.7%) males], 107 (71.3%) had ischemic stroke in anterior circulation including 56 (37.3%) patients with acute cerebral infarction, 51 (34.0%) patients with acute lacunar infarction and 43 (28.7%) patients with TIA. Among the 150 patients who suffered from cerebrovascular symptoms at baseline, 99 (66.0%) had culprit lesions in extracranial carotid arteries, 48 (32.0%) had culprit lesions in intracranial arteries and the culprit lesions were uncertain in 3 (2.0%) patients. The clinical data are detailed in Table 2. Patients with subsequent vascular events had significantly greater age (64.9 ± 10.4 vs. 60.6 ± 12.2 years old, *P* = 0.047) and NIHSS score (5.6 ± 4.3 vs. 3.3 ± 3.0, *P* = 0.003) compared to those without subsequent vascular events. No significant differences can be observed in other clinical characteristics between these two patient groups (all *P* > 0.05).

**Table 2:** Baseline and follow-up clinical characteristics of the study population (*n* = 150)

	Mean ± SD, or *n* (%)		
	Patients with vascular events (*n* = 41)	Patients without vascular events (*n* = 109)	P value
Baseline clinical characteristics			
Age, years	64.9 ± 10.4	60.6 ± 12.2	0.047
Sex, male	28 (68.3)	81 (74.3)	0.461
BMI, kg/m^2^	25.6 ± 3.3	25.5 ± 3.4	0.868
History of smoking	24 (58.5)	67 (61.5)	0.743
History of hypertension	28 (68.3)	79 (72.5)	0.614
History of diabetes	7 (17.1)	35 (32.1)	0.068
History of hyperlipidemia	16 (39.0)	59 (54.1)	0.099
History of coronary heart disease	5 (12.2)	12 (11.0)	0.839
History of stroke	20 (48.8)	38 (34.9)	0.119
Systolic blood pressure, mm Hg	147.6 ± 23.2	144.7 ± 24.0	0.223
Diastolic blood pressure, mm Hg	88.6 ± 13.5	86.6 ± 16.2	0.236
HDL, mmol/L	1.1 ± 0.3	1.2 ± 0.6	0.374
LDL, mmol/L	2.5 ± 1.4	2.9 ± 1.4	0.096
Total cholesterol, mmol/L	4.2 ± 1.3	4.4 ± 1.2	0.398
Triglycerides, mmol/L	1.7 ± 1.4	1.7 ± 1.0	0.646
NIHSS score	5.6 ± 4.3	3.3 ± 3.0	0.003
Drug treatment during follow-up			
Antihypertension	17 (41.5)	49 (45.0)	1.000
Lipid-lowering	33 (80.5)	86 (78.9)	0.203
Anticoagulation	22 (53.7)	63 (57.8)	0.064
Antiplatelet	21 (51.2)	39 (35.8)	0.368

*BMI* body mass index, *HDL* high-density lipoprotein, *LDL* low-density lipoprotein, *NIHSS* National Institutes of Health Stroke Scale

### Baseline characteristics of cerebrovascular atherosclerosis on CMR imaging

Of 150 patients, 38 (25.3%) had stenosis < 50%, 4 (2.7%) had stenosis in the range of 50–69%, and 15 (10.0%) had stenosis ≥70% in extracranial carotid arteries, respectively. In this study population, the prevalence of plaque, calcification, LRNC and IPH was 88.0% (132/150), 44.0% (66/150), 66.0% (99/150), and 17.3% (26/150) in extracranial carotid arteries, respectively. In intracranial arteries, 48 (32.0%) patients had stenosis < 50% and 48 (32.0%) patients had stenosis ≥50%, respectively. The distribution of intracranial artery stenosis is detailed in Additional file 1: Table S1. The comparison results of cerebrovascular atherosclerosis between patients with and without subsequent vascular events are summarized in Table 3. Patients with subsequent vascular events had significantly greater prevalence of intracranial stenosis (80.5% vs. 57.8%, *P* = 0.010) and extracranial carotid LRNC (82.9% vs. 59.6%, *P* = 0.007) compared to those without subsequent vascular events. No significant differences can be found in prevalence of other plaque compositions (all *P* > 0.05).

**Table 3:** Characteristics of intracranial artery and extracranial carotid artery atherosclerosis

	Mean ± SD, or *n* (%)		P value
	Patients with vascular events (*n* = 41)	Patients without vascular events (*n* = 109)	
Extracranial carotid artery			
Maximum wall thickness, mm	3.3 ± 1.7	3.0 ± 1.4	0.316
Presence of stenosis			
Intracranial stenosis	33 (80.5)	63 (57.8)	0.010
Extracranial carotid stenosis	17 (41.5)	40 (36.7)	0.592
Presence of extracranial carotid plaque components			
Calcification	22 (53.7)	44 (40.4)	0.144
Lipid-rich necrotic core	34 (82.9)	65 (59.6)	0.007
Intraplaque hemorrhage	9 (22.0)	17 (15.6)	0.359

### Association between co-existing plaques and subsequent vascular events

**Fig. 1:**
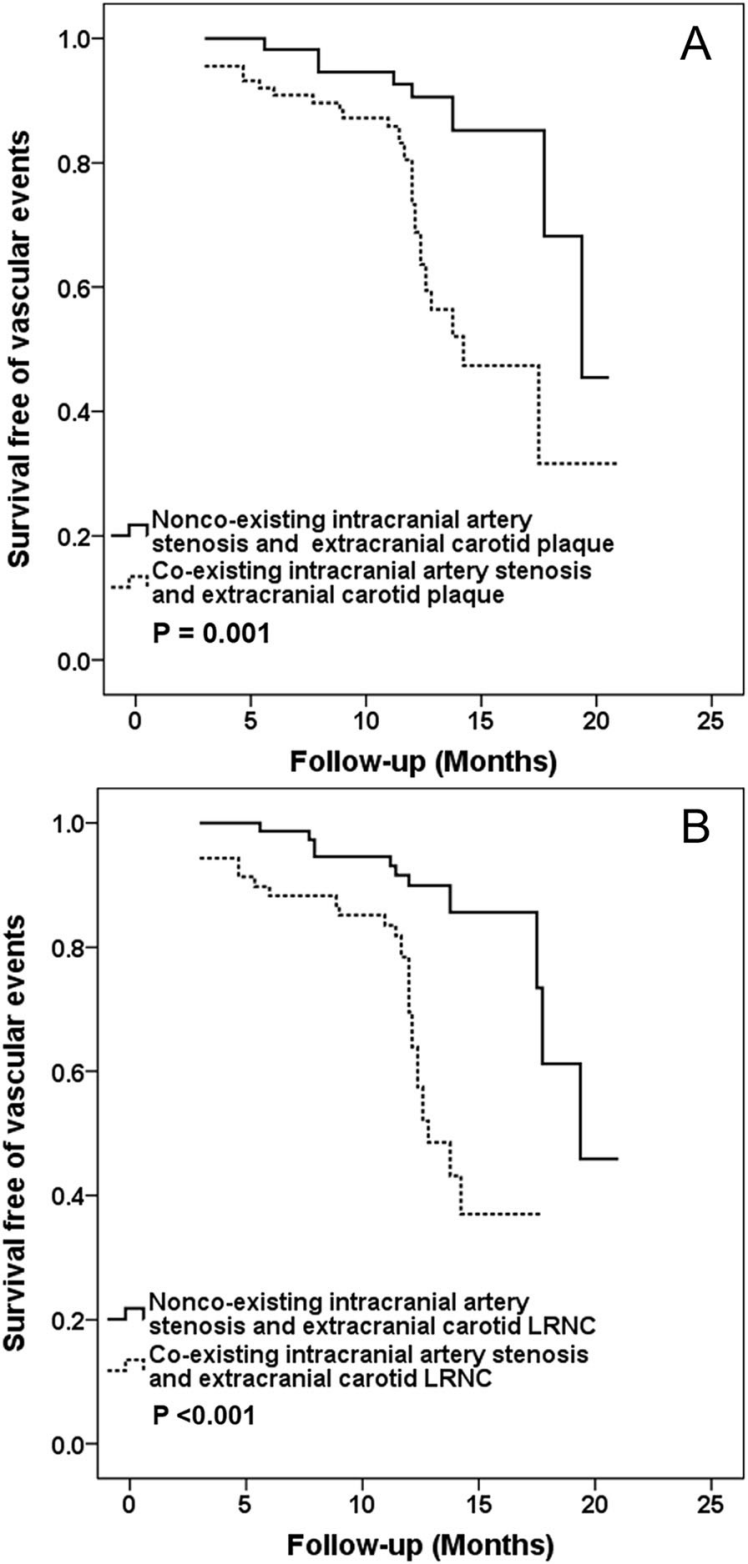
Kaplan-Meier analysis of survival free of vascular eventsin co-existing diseases. Kaplan-Meier analysis of survival free of vascular events in the nonco-existing intracranial artery stenosis and extracranial carotid plaque and co-existing intracranial artery stenosis and extracranial carotid plaque with up to 21 months follow-up (**a**). Kaplan-Meier analysis of survival free of vascular events in the nonco-existing intracranial artery stenosis and extracranial carotid lipid rich necrotic core (LRNC) and co-existing intracranial artery stenosis and extracranial carotid LRNC with up to 21 months follow-up (**b**). The X-axis represents the time of follow-up in months. The Y-axis represents the proportion of patients who were survival free of vascular events

**Fig. 2:**
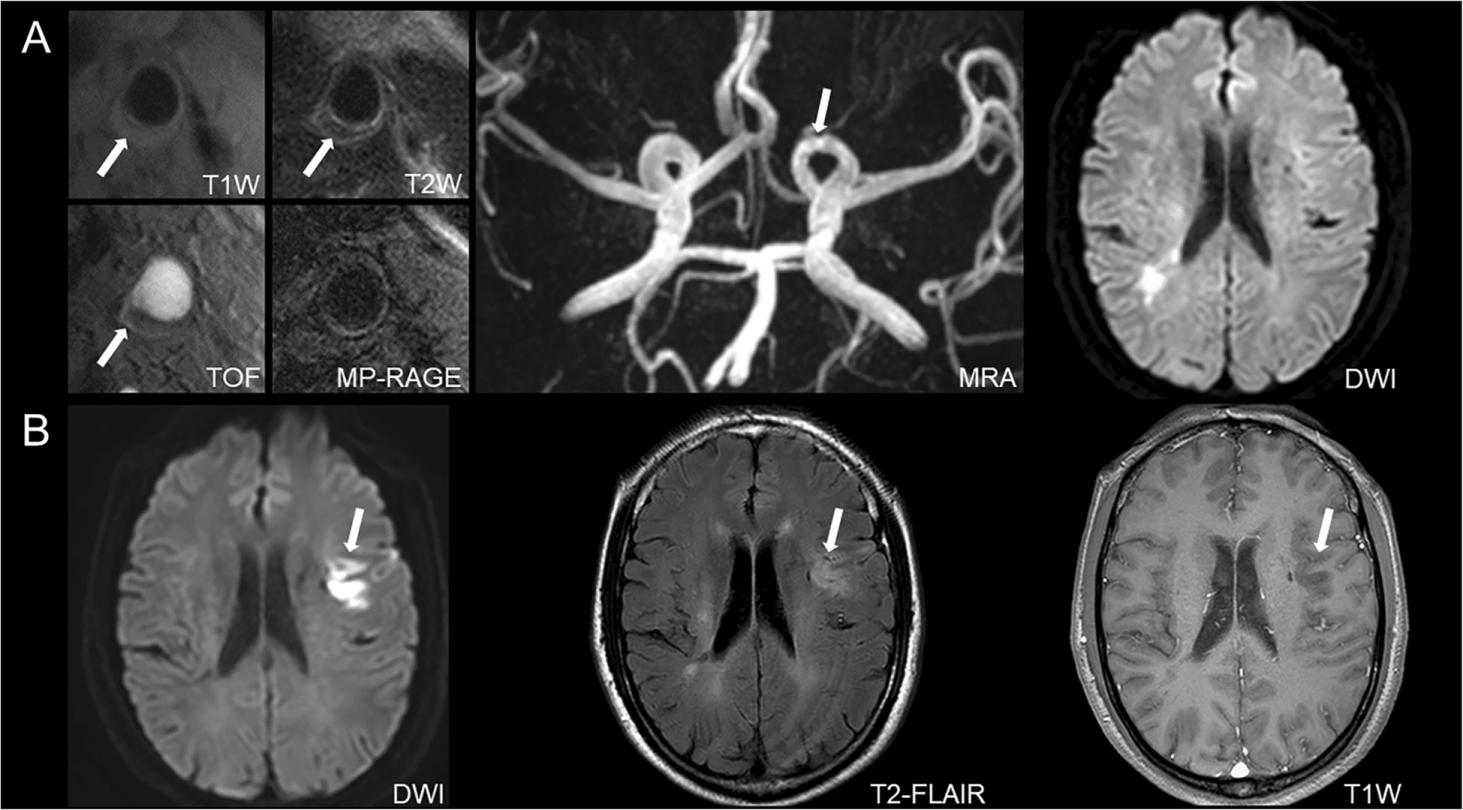
Patient with co-existing intracranial artery stenosis and extracranial carotid LRNC developed recurrent stroke. An example for a 63 years old male patient who had atherosclerotic plaque with lipid-rich necrotic core in the left extracranial carotid artery bifurcation (white arrows on A [T1 weighted (T1W), T2 weighted (T2w) and time-of-flight (TOF)]) and intracranial artery stenosis (white arrow on A [MRA]). After a year of follow-up, a new acute infarction was detected in the left hemisphere (white arrows on B [diffusion weighted imaging (DWI), T2-FLAIR and T1w])

Of the 150 patients, 96 (64.0%) had intracranial stenosis, of which 89 (59.3%), 45 (30.0%), 70 (46.7%), and 19 (12.7%) had co-existing plaque, calcification, LRNC, and IPH in carotid arteries, respectively. After baseline examination, some patients with carotid endarterectomy (*n* = 8) were excluded from our study and the remaining patients (*n* = 150) received the medical treatment during follow-up. During the median follow-up time of 12.1 months, 41 (27.3%) patients were suffered from subsequent vascular events including 15 (10.0%) patients with recurrent ischemic stroke diagnosed by CMR and 26 (17.3%) patients with recurrent vascular events determined by medical records during follow-up. Of 41 patients with recurrent vascular events, 17 had ischemic strokes in the territory of anterior circulation (11 acute cerebral infarcts and 6 acute lacunar infarcts), 19 had TIAs, and 5 had acute coronary syndromes. Table 4 presented the results on Cox regression analysis. Univariate Cox regression analysis showed that co-existing intracranial stenosis and carotid plaque (HR, 3.57; 95% CI, 1.63–7.82; *P* = 0.001) and LRNC (HR, 4.47; 95% CI, 2.15–9.27; *P* < 0.001) were significantly associated with subsequent vascular events. After adjusted for baseline confounding factors including age, sex, BMI, history of stroke, diabetes, hypertension, hyperlipidemia, coronary heart disease, smoking, NIHSS, carotid stenosis and treatment procedures including antihypertension, lipid-lowering, anticoagulation and antiplatelet during follow-up, these associations remained statistically significant (co-existing intracranial stenosis and carotid plaque: HR = 5.12, 95% CI 1.36–19.24, *P* = 0.016; co-existing intracranial stenosis and carotid LRNC: HR = 8.12, 95% CI 2.41–27.31, *P* = 0.001). The subsequent vascular events were not significantly associated with co-existing intracranial stenosis and carotid IPH and calcification (all *P* > 0.05). Since only few cases did not have carotid plaques, the HR value of presence of extracranial carotid plaque for predicting subsequent vascular events was not calculated due to the potential issue of overfitting. The prevalence of cumulative vascular events for patients with co-existing intracranial artery stenosis and extracranial carotid plaque at 5, 10, 15 and 20 months was 4.0% (6/150), 7.3% (11/150), 21.3% (32/150) and 22.0% (33/150), respectively. The prevalence of cumulative vascular events for patients with co-existing intracranial artery stenosis and extracranial carotid LRNC at 5, 10, 15 and 18 months was 4.0% (6/150), 6.7% (10/150), 20.0% (30/150) and 20.0% (30/150), respectively. Kaplan-Meier curves for the incidence of subsequent vascular events showed that event-free survival was significantly higher for patients in the nonco-existing intracranial artery stenosis and extracranial carotid plaque group than ones in the co-existing intracranial artery stenosis and extracranial carotid plaque group (*P* = 0.001; Fig. 1a). Kaplan-Meier curves for the incidence of subsequent vascular events showed that event-free survival was significantly higher for patients in the nonco-existing intracranial artery stenosis and extracranial carotid LRNC group than ones in the co-existing intracranial artery stenosis and extracranial carotid LRNC group (*P* < 0.001; Fig. 1b). Figure 2 is an example for patient who had co-existing intracranial and extracranial carotid LRNC developed subsequent ischemic stroke after one-year follow-up.

**Table 4:** Cox regression hazard models of risk factors for vascular events

	Vascular events					
	Univariate Regression			Multivariate Regression*		
	HR	95% CI	P value	HR	95% CI	P value
Extracranial carotid artery						
Maximum wall thickness, mm	1.06	0.88–1.28	0.542	0.91	0.67–1.23	0.526
Presence of calcification	1.40	0.76–2.60	0.280	1.04	0.32–3.33	0.954
Presence of LRNC	3.65	1.60–8.35	0.002	4.26	0.96–18.80	0.056
Presence of IPH	1.25	0.60–2.64	0.552	0.57	0.09–3.60	0.552
Presence of stenosis	1.16	0.62–2.16	0.647	–	–	–
Intracranial artery						
Presence of stenosis	2.73	1.25–5.97	0.012	5.95	0.97–36.38	0.054
Co-existing plaques						
Intracranial stenosis and extracranial carotid plaque	3.57	1.63–7.82	0.001	5.12	1.36–19.24	0.016
Intracranial stenosis and extracranial carotid calcification	1.74	0.94–3.24	0.078	2.95	0.76–11.42	0.117
Intracranial stenosis and extracranial carotid LRNC	4.47	2.15–9.27	< 0.001	8.12	2.41–27.31	0.001
Intracranial stenosis and extracranial carotid IPH	1.67	0.77–3.63	0.194	1.44	0.17–12.39	0.738

*HR* hazard ratio, *CI* confidence interval, *LRNC* lipid-rich necrotic core, *IPH* intraplaque hemorrhage. *Adjusted for baseline confounding factors including age, sex, BMI, history of stroke, diabetes, hypertension, hyperlipidemia, coronary heart disease, smoking, NIHSS score, and carotid stenosis and treatment procedures during follow-up including antihypertension treatment, lipid-lowering treatment, anticoagulation treatment and antiplatelet treatment

### Reproducibility

For the intra-observer agreement in identification of the presence of carotid plaque, calcification, LRNC and IPH, the kappa value was 1.00, 0.78, 0.80 and 0.89 (all *P* < 0.001), respectively. For the inter-observer agreement in assessing of the presence of carotid plaque, calcification, LRNC and IPH, the kappa value was 1.00, 0.87, 0.80 and 0.78 (all *P* < 0.001), respectively. The intra-observer ICC of carotid Max WT, luminal stenosis and intracranial artery stenosis was 0.98 (95% CI, 0.94–0.99, *P* < 0.001), 0.96 (95% CI, 0.90–0.98, *P* < 0.001) and 0.98 (95% CI, 0.94–0.99, *P* < 0.001), respectively. The inter-observer ICC was 0.98 (95% CI, 0.96–0.99, *P* < 0.001), 0.93 (95% CI, 0.84–0.97, *P* < 0.001) and 0.98 (95% CI, 0.94–0.99, *P* < 0.001) for carotid Max WT, luminal stenosis and intracranial artery stenosis, respectively.

## Discussion

This study investigated the relationship between co-existing intracranial stenosis and extracranial vulnerable plaques and subsequent vascular events utilizing CMR vessel wall imaging. We found that the co-existing intracranial arteries stenosis and extracranial carotid plaque or LRNC determined by CMR vessel wall imaging was independently associated with subsequent vascular events. Our findings suggest that co-existing intracranial stenosis with extracranial carotid artery plaque vulnerable features, such as LRNC, have higher predictive value for occurrence of subsequent vascular events compared to the measurement in single vascular bed.

In the present study, 59.3% of symptomatic patients had co-existing extracranial carotid artery plaque and intracranial artery stenosis. Our findings are in line with previous reports with the prevalence of co-existing plaques ranging from 42.2 to 64% in symptomatic patients [4, 5, 13]. Different from previous studies, Xu et al. [10] utilized CMR vessel wall imaging to detect atherosclerotic disease and found that the prevalence of co-existing intracranial and extracranial plaque was 77.6% in symptomatic patients. In the present study, the presence of extracranial carotid plaque was also determined by CMR vessel wall imaging. It is well established that CMR vessel wall imaging can yield more lesion detection compared with angiographic imaging approach because it provides direct information of the lesions in the arterial wall [14, 15]. The prevalence of co-existing cerebrovascular atherosclerotic plaques in the present study was lower than that in Xu’s study [10] which might be due to different study population. In Xu’s study [10], all the patients had plaque in at least one side of carotid arteries. The presence of co-existing plaques in intra- and extra-cranial vascular beds further demonstrated that the atherosclerosis can develop systemically.

We found that the co-existing intracranial artery stenosis and extracranial carotid plaque determined by CMR vessel wall imaging was independently associated with subsequent vascular events. The co-existing intracranial and extracranial atherosclerosis may represent a systematic burden of atherosclerotic disease. Similar to our findings, previous studies also reported that patients with co-existing plaques had higher risk of cardiovascular or cerebrovascular events. A prospective study in which patients were followed-up up to 76 months by Man et al. [16] showed that the presence of concurrent intracranial and extracranial stenoses measured by CMRA was independent predictors of poor outcomes. Hoshino et al. [17] investigated the vascular prognosis for patients with acute ischemic stroke with intracranial and extracranial plaques after 4 years of follow-up and found that among patients with intracranial atherosclerosis, concurrent stenosis in the extracranial carotid artery (23.4% vs. 9.0%; *P* = 0.08; adjusted HR = 2.12) increased the major adverse cardiovascular events risk. These findings indicate that the co-existing intracranial and extracranial plaques can predict future vascular events.

The co-existing intracranial artery stenosis and extracranial carotid LRNC were independently associated with subsequent vascular events. This study is one of the first to investigate the predictive value of co-existing carotid vulnerable plaque and intracranial stenosis for future vascular events. The finding is expected because the association between carotid LRNC and cerebrovascular events has been well evidenced in previous studies. Takaya et al. [18] demonstrated that among patients who initially had an asymptomatic 50 to 79% carotid stenosis, larger maximum percentage of LRNC (HR for 10% increase, 1.6; *P* = 0.004) by CMR was associated with the occurrence of subsequent cerebrovascular events. Another CMR study by Kwee et al. [19] claimed that the presence of LRNC (HR, 3.20; 95% CI, 1.08–9.50; *P* = 0.036) was associated with the recurrence of clinical cerebrovascular ischemic events in TIA and stroke patients with carotid atherosclerosis. Investigators also found that there was significant correlation between carotid LRNC and cardiovascular events. Zavodni et al. [20] showed that a lipid core at CMR imaging confers increased risk for subsequent cardiovascular events in asymptomatic individuals. Sun et al. [21] reported that high lipid content (HR per one-standard-deviation increase in % lipid core volume: 1.57, *P* = 0.002) in carotid plaques was strongly associated with the cardiovascular outcomes. The LRNC within atherosclerotic plaque, particularly its size, has been believed to be a key feature of vulnerable plaque [22, 23]. The increasement of the LRNC size may be contributed to the changes of cholesterol from the liquid to solid crystal state and will stimulate fibrous cap extension and thinning or even rupture [24]. In the presence study, we used CMR vessel wall imaging to identify not only LRNC but also IPH. However, we did not find significant correlation of co-existing intracranial stenosis and extracranial carotid IPH with vascular events, though this compositional feature is associated with plaque vulnerability [22]. The potential reason may be the smaller number of co-existing IPH (*n* = 26) in this study population. Our findings indicate that the assessment of co-existing intracranial and extracranial atherosclerosis needs to not only pay attention to presence of plaque but also characterize plaque compositional features, particularly lipid-rich components. Our findings suggest that patients with co-existing lesions may have higher risk of developing future vascular events and intense intervention medication may be warranted for these patients.

## Limitations

The present study has several limitations. First, the intracranial atherosclerosis was identified by TOF CMRA that lacks of vessel wall information. TOF CMRA may yield underestimation of intracranial plaques when there is positive remodeling in the vessel wall [25]. Future studies utilizing vessel wall imaging to characterize plaque features of intracranial arteries are warranted. Second, the follow-up time was short and the usefulness of co-existing plaque in predicting long-term outcomes cannot be determined. Third, in the present study, the size of the intraplaque compositions of co-existing intracranial and extracranial artery atherosclerosis was not assessed. Previous evidences showed that the size of LRNC [18, 21] and IPH [18] was associated with future vascular events. Fourth, the longitudinal coverage of current carotid imaging protocol is 32 mm centered to carotid bifurcation. This coverage is limited for atherosclerotic lesions occurring in more proximal or distal segments of carotid arteries. The 3D vessel wall imaging techniques [26] with larger longitudinal coverage are suggested to be utilized in future studies. Finally, the HR value of extracranial carotid plaque in predicting vascular eventswas not analyzed because atherosclerotic plaques were present in most of the cases of our study population. Future studies will increase the sample size to enhance such statistical power.

## Conclusions

The co-existing cerebrovascular atherosclerotic diseases, particularly co-existing carotid lipid-rich necrotic core and intracranial stenosis, are independent predictors for subsequent vascular events. Our findings suggest that it is valuable to characterize the compositional features of co-existing intracranial and extracranial carotid atherosclerosis in stratifying the risk of subsequent vascular events.

## Supplementary information

**Additional file 1 Table S1**. The distribution of intracranial artery stenosis.

## Data Availability

The datasets used and/or analyzed during the current study are available from the corresponding author [X.Z.] on reasonable request. The data are not publicly available due to them containing information that could compromise research participant privacy/consent.

## Abbreviations

BMI: Body mass index
CI: Confidence interval
CMR: Cardiovascular magnetic resonance
CMRA: Cardiovascular magnetic resonance angiography
CTA: Computed tomographic angiography
DWI: Diffusion weighted imaging
FLAIR: Fluid attenuated inversion recovery
HR: Hazard ratio
ICC: Intra-class correlation coefficient
IPH: Intraplaque hemorrhage
LRNC: Lipid-rich necrotic core
Max WT: Maximum wall thickness
MERGE: Motion sensitized driven Equilibrium prepared Rapid Gradient Echo
MP-RAGE: Magnetization-prepared rapid acquisition gradient echo
NASCET: North American Symptomatic Carotid Endarterectomy Trial
T1W: T1 weighted
T2W: T2 weighted
TIA: Transient ischemic attack
TOF: Time-of-flight
