# Supplementary material for: Co-existing cerebrovascular atherosclerosis predicts subsequent vascular event: a multi-contrast cardiovascular magnetic resonance imaging study
Source: J Cardiovasc Magn Reson. 2020 Jan 13;22:4. doi: 10.1186/s12968-019-0596-6 (PMC6956475; doi:10.1186/s12968-019-0596-6)
Supplement: Supplementary file 1 — Additional file 1 Table S1. The distribution of intracranial artery stenosis. [file 12968_2019_596_MOESM1_ESM.docx]

**Supplemental Table**

**Supplemental Table.** The distribution of intracranial artery stenosis.

| Location | Number of stenosis lesions (%) | | |
| --- | --- | --- | --- |
|  | Left | Right | Total |
| Intracranial internal carotid artery | 35 (23.3) | 41 (27.3) | 76 (50.6) |
| M1 segment of middle cerebral artery | 41 (27.3) | 44 (29.3) | 85 (56.6) |
| A1 segment of anterior cerebral artery | 34 (22.7) | 34 (22.7) | 68 (45.4) |
| Total | 110 (73.3) | 119 (79.3) | 229 |
